# Influence of Ultrasonication and UV-C Processing on the Functional Characteristics and Anticarcinogenic Activity of Blackthorn Vinegar

**DOI:** 10.1021/acsomega.4c05363

**Published:** 2024-08-16

**Authors:** Sıla Barut Gök, Seydi Yıkmış, Okan Levent, Esra Bozgeyik, Kerem İlaslan, Vahide Gizem Aydın

**Affiliations:** † Department of Food Technology, Tekirdağ Namık Kemal University, Tekirdağ 59830, Turkey; ‡ Department of Food Engineering, Faculty of Engineering, Inonu University, Malatya 44280, Turkey; § Department of Medical Services and Techniques, Health Services Vocational School, Adıyaman University, Adıyaman 02040, Turkey; ∥ Department of Gastronomy and Culinary Arts, School of Applied Sciences, Bahçeşehir University, İstanbul 34353, Turkey; ⊥ Department of Nutrition and Dietetics, School of Health Sciences, Tekirdağ Namık Kemal University, Tekirdağ 59030, Turkey

## Abstract

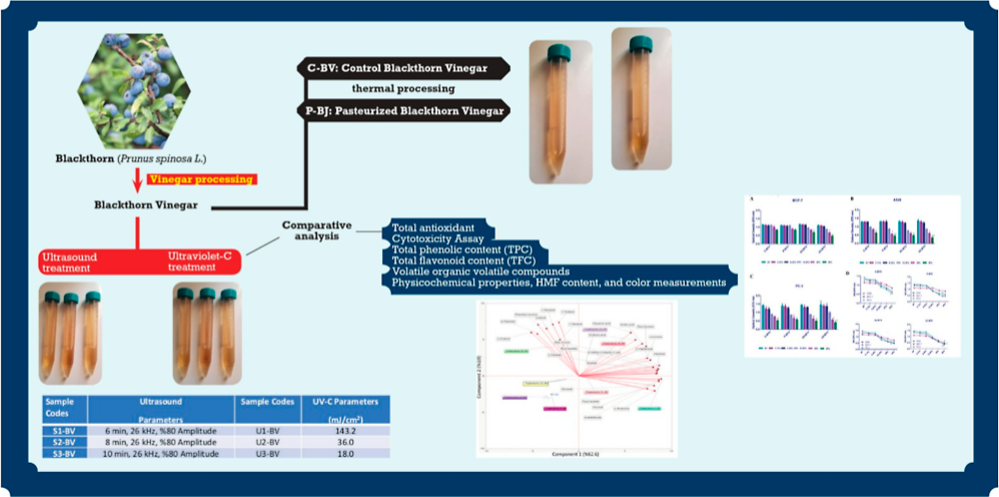

In recent years, consumer trends have been changing toward fresh food products such as fruit juice, vinegar, etc. that are a good source of bioactive components, high nutritional characteristics, and beneficial microorganisms. Blackthorn (*Prunus spinosa* L.) vinegar (BV) is one of these nutritious foods. The study aims to examine the efficacy of ultraviolet-C (UV-C) light applied by a modified reactor and ultrasonication on bioactive compounds (total phenolic, total flavonoid, ascorbic acid content, and antioxidant activity) of traditionally produced BV. Furthermore, the volatile organic compound (VOC) profile, hydroxymethylfurfural (HMF) content, cytotoxicity properties, and color were assessed. UV-C light and ultrasonication processes enriched most bioactive components, but these methods did not significantly improve ascorbic acid (*p* > 0.05) compared to pasteurization. Twenty-seven volatile compounds were analyzed in order to determine the VOC profile. As a result, thermal and nonthermal methods were found to affect the profile significantly (*p* < 0.05). No significant differences were detected in total soluble solids (4.70–4.77), titratable acidity (3.81–3.87), and pH (3.39–3.41) values. The anticarcinogenic activities of UV-C-treated BVs were more significant than others. Nonthermal treatments were generally better than pasteurization in maintaining and enriching the quality of BV. In this study, UV-C light and ultrasonication technology can be used as an alternative to traditional thermal techniques to improve the quality of BV.

## 1 Introduction

Medicinal plants are the primary sources of natural bioactive compounds. In recent years, there has been growing interest in components of functional products that are active in the prevention of numerous chronic diseases such as cardiovascular disorders.^1^ Ethnobotanical knowledge constitutes a substantial guide to finding functional products with related activity.^1,2^ Recently, many studies have focused on plant and microbial extracts, essential oils, secondary metabolites, and newly synthesized molecules as potential antimicrobial agents.^3^

The popularity of fresh food products is increasing, with many nutrition researchers encouraging the regular consumption of bioactive substances. A promising plant species that has come to the fore lately is *Prunus spinosa* L. (*P. spinosa* L), known as blackthorn, jackal plum, or güvem in Turkish. It is a perennial thorny shrub that grows wild in uncultivated areas of Europe, Western Asia, and the Mediterranean. Blackthorn has been used as an anti-inflammatory and antiseptic and for the treatment of coughs through phototherapy.^4^ High tannin content improves these fruits in terms of antioxidant, antibacterial, antifungal, anti-inflammatory, and antidiabetic properties.^5−8^ Blackthorn fruits can be used for making products like jam and beverages because of their pungent taste and high anthocyanin content. However, it also has the potential for use as a new food/dressing or food additive, such as vinegar, which can be considered a healthy alternative for consumers.^8^

Vinegar has been produced and widely used worldwide for thousands of years. Vinegar is a fermented food seasoning with abundant microbial resources and local characteristics.^9,10^ It is an acidic liquid produced by a two-stage bioprocess. In the first stage, yeasts, normally strains of *Saccharomyces cerevisiae*, convert fermentable sugars into ethanol. In the second stage, ethanol is oxidized to acetic acid by the bacteria of the genus *Acetobacter*.^11^ Various reports reveal many positive effects on health, thanks to the bioactive components it contains.^12−15^ However, the number of studies in which characterization of vinegars using different raw materials or traditionally produced vinegars is fairly limited. Phenolic components strongly depend on the production process and the raw material used in its production and affect the antimicrobial and antioxidant characteristics of vinegar.^16,17^

The popularity of fresh-like food products is increasing with the recommendations to promote the regular consumption of bioactive components by many nutritional researchers. Although the thermal treatment is addressed to provide microbial inactivation and extend the shelf life, the nutrient contents and sensorial characteristics in fresh-like products are quite susceptible to deterioration due to influencing factors like heat during processing and storage.^18,19^ Therefore, increasing consumer demand for minimally processed foods^20^ and the nutrient loss related to the conventional preservation methods^21,22^ direct the trend toward nonthermal or novel technologies. However, nonthermal techniques are used as an alternative to thermal methods such as pasteurization in various foods, including fruit juices, vinegar, etc.^15,23−28^

Ultrasound or ultrasonic wave technology is defined as pressure waves with a commercially available frequency of 20 or 40 kHz,^29^ which cause physical and chemical alterations in biological structures.^30^ Ultrasound treatment is widely recognized as a technology that is environmentally friendly, innovative, cost-effective, rapidly developing, and scalable.^31^ It has been shown that the use of ultrasound can enrich the bioactive components of food products such as juices and vinegar.^32−35^ UV-C light has been used in the food industry for reducing the level of pathogens in meat, fish, poultry, ready-to-eat produce, minimally processed fresh foods, dairy products,^24,36^ fruit juices,^37−39^ etc. The primary mechanism of the UV-C energy on inactivation of microorganisms is due to the formation of pyrimidine dimers, which prevent replication and provide inactivation of microorganisms.^40^ Both techniques are considered safe, nontoxic, cost-effective, nutrient-enriching, and environmentally friendly for food processing.^41−43^ Results of recent studies demonstrated that UV-C light and US have the potential as an alternative to thermal pasteurization/treatments without any nutritional, sensorial, or physicochemical changes in the quality of the liquid products.^44−46^

In this study, a designed reactor^39,47^ of UV-C and ultrasonication were used to evaluate the changes in bioactive compounds [total phenolic content (TPC), total flavonoid content (TFC), total monomeric anthocyanin and ascorbic acid content, and antioxidant activity] of traditionally produced BV. In addition, the effects of treatments on the volatile organic compound (VOC) profile, HMF content, anticancer properties, and color were compared to pasteurized samples. To our knowledge, there is no study in the literature regarding the efficiency of UV-C or the comparison of UV-C light and ultrasound treatment on the quality characteristics and anticarcinogenic activity of blackthorn vinegar or the traditional vinegar produced using different raw materials.

## 2 Materials and Methods

### 2.1 Preparation of BV

Blackthorn fruits were collected from the Türkiye/Tekirdağ region for vinegar production. The rotten fruits were separated from the collected ones. The seeds of the fruits were removed, and the flesh was crushed. The crushed fruits were mixed with deionized water (1:1 w/w), and 15% pine honey was added to the mixture as a carbohydrate source for fermentation. The BV was then produced using a traditional method described in a previous study.^12^ The BV samples were stored at −20 ± 1 °C in 100 mL sterile glass jars until the analysis. The traditionally produced BV was used as a control (C-BV). Experiments were performed in triplicate.

### 2.2 Pasteurization, Ultrasonication, and UV-C Treatments

The pasteurization process was carried out with the method used in the previous study.^12^ The pasteurized BV was named P-BV. 100 mL of vinegar samples were processed using a 200 W ultrasonic processor (Hielscher Ultrasonics, model UP200 St, Berlin, Germany) at a frequency of 26 kHz. The amplitude, time parameters, and sample codes are given in Table 1. An ice bath was used to control temperature during the ultrasonication process. Samples were stored at −18 °C until analysis.

**Table 1:** Parameters of Ultrasonication and UV-C Treatment of the BV

sample codes	ultrasound parameters	sample codes	UV-C parameters (mJ/cm^2^)
S1-BV	6 min, 26 kHz, % 80 amplitude	U1-BV	143.2
S2-BV	8 min, 26 kHz, % 80 amplitude	U2-BV	36.0
S3-BV	10 min, 26 kHz, % 80 amplitude	U3-BV	18.0

The modified UV-C reactor included a grooved stainless-steel semicircle flow path positioned around a quartz tube containing a UV-C source.^39^ Three different flow rates through the reactor were adjusted to obtain different doses with a peristaltic pump. The UV-C dose (*D*) was calculated according to eq 1, where *I*_avg_ and *t* express the UV radiation intensity and the exposure time, respectively. The average intensity (*I*_avg_) was calculated as given in eq 2 by using the incident intensity (*I*_o_, 22.3 mW cm^–2^).^48^*A*_e_ (1/cm) and *L* (cm) are the absorption coefficients detected at a 254 nm wavelength and the path length of the cuvette, respectively. The slope of the absorbance versus dilution factor curve is used to estimate the absorption coefficient (*A*_e_, 13.63 1/cm) of the vinegar sample. UV-C doses and sample codes are also given in Table 1.
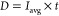
1
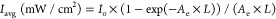
2

### 2.3 Bioactive Compounds

The TPC was analyzed according to the method based on the reaction between the Folin–Ciocalteu reagent and phenolics of BV following the procedure described by Singleton and Rossi (1965).^100^ The TPC was expressed as milligrams of gallic acid equivalent per L (mg of GAE/L). The TFC was determined by a colorimetric method according to Zhishen et al. (1999).^56^ The TFC was calculated and expressed as milligrams of catechin equivalent per mL (mg CE/mL). The antioxidant capacity was evaluated using the method based on the DPPH radical scavenging capacity with some modifications and expressed as mg Trolox equivalent antioxidant capacity (TEAC) per mL (mg TEAC/mL).^57^ Also, the cupric reducing antioxidant capacity (CUPRAC) method was carried out according to Apak et al. (2006) to evaluate the antioxidant capacity.^58^ The ascorbic acid content was determined by the method of Ordóñez-Santos and Vázquez-Riascos (2017), and the results were calculated as mg/100 mL.^60^ The pH-differential method was performed to calculate the total monomeric anthocyanin content (TAC).^59^

**Figure 1:**
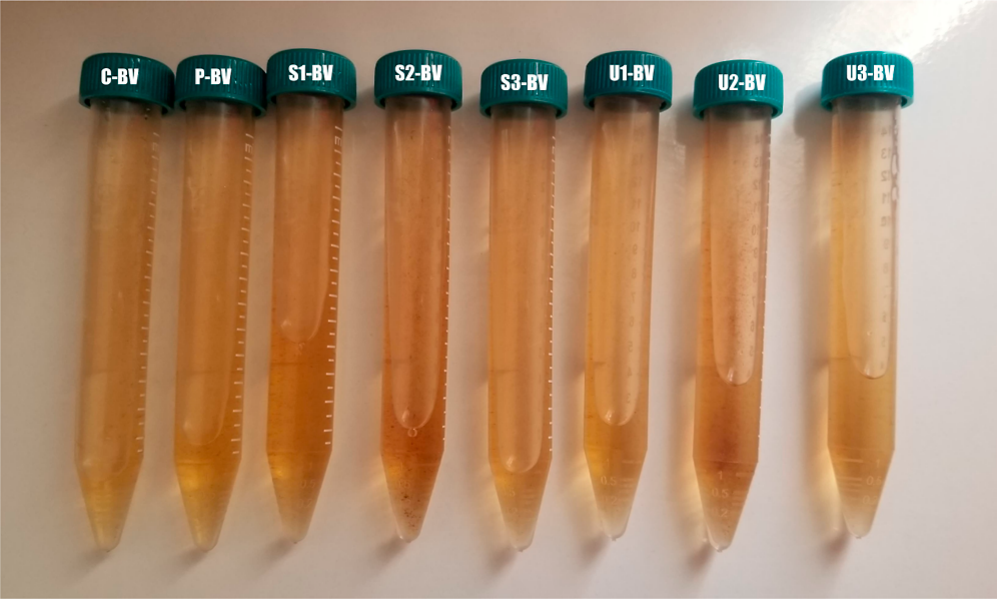
Prepared samples and codes of blackthorn vinegar.

The ultrasonication parameters and UV-C doses for the research were chosen according to the results of the previous studies performed with the current modified UV-C reactor and ultrasonication process in liquids such as various fruit juices and vinegar.^28,39,49,50^ The prepared samples are shown in Figure 1 with their codes (Figure 1). The main target of the current and previous studies using UV-C and US is to practice minimal conditions in order to minimize processing of the food product, maximize nutritional and sensorial characteristics, and maintain its safety. Although better results are determined with the ultrasonication process compared to heat pasteurization, ultrasound technology is reported to be more efficient in combination with other emerging techniques^51^ rather than the treatment alone. In addition to this, the sensorial and nutritional quality of food products may be adversely affected by treatment with high power levels of ultrasound.^52,53^ Therefore, a combination of US with other nonthermal techniques such as UV-C, high pressure, and pulsed electric field is often performed^51,52,54,55^ such as the current study.

### 2.4 Cytotoxicity Assay

The cytotoxic effects of the BV obtained by different techniques were assayed in A549 lung cancer cells (ATCC, CCL-185), MCF-7 breast cancer cells (ATCC, HTB-22), and prostate cancer cells PC-3 (ATCC, CRL-1435). Cells were grown in Dulbecco’s modified Eagle medium containing 10% fetal calf serum and 5% streptomycin/penicillin (Sigma-Aldrich, Germany) in an incubator containing 5% CO_2_ at 37 °C. Cells reaching a certain density were trypsinized and seeded on 96-well plates as 1 × 10^4^ cells per well. Following 24 h of incubation, different concentrations (5, 25, 12.5, 6.25, and 3.125%) of vinegar samples were applied. Then, plates were washed with 1× PBS and incubated with 100 μL of 3-(4,5-dimethylthiazol-2-yl)-2,5-diphenyltetrazolium bromide (MTT) (1:1 mg/mL) for 45–60 min. MTT was dissolved with dimethyl sulfoxide (DMSO) and read at a 570 nm wavelength in a Multiscan GO microplate reader (Thermo Scientific, USA). Cytotoxicity and % inhibitions were calculated according to OD values.^12,42^

### 2.5 VOC Profiles

Volatiles of the BV were determined by divinylbenzene-carboxy-polydimethylsiloxane solid-phase microextraction (SPME) fiber assembly (50 μm DVB layer/30 μm CAR/PDMS layer; 2 cm fiber length; Supelco, USA). A 3 mL portion of the vinegar sample was immediately transferred to vials, followed by 10 μL of an internal standard containing 81 mg/kg 2-methyl-3-heptanone and 2-methyl pentanoic acid (for volatile organic acids) in methanol as an internal standard. The vials were placed on a heater at 40 °C for 30 min to allow the volatiles to accumulate in the headspace. The fiber was then injected into the vial to absorb the volatiles for 30 min. A gas chromatography–mass spectrometry system (GC–MS) (Shimadzu GC-2010, QP-2010, Japan) was used to desorb the extracted volatiles at 250 °C. Separation was performed with the DB-Wax column (60 m × 0.25 mm × 0.25 mm). The volatile content of the vinegar sample was identified by a retention index using the *n*-alkane series (C10–C26) under the same conditions. WILEY8 and NIST05 mass spectral libraries were used for the identification.

### 2.6 HMF and Color Measurements

LeBlanc et al. (2009) described a method for the HMF determination. The absorbance of the vinegar was analyzed at 550 nm when the intensity of the color reached a maximum level.

Color analysis was performed using a colorimeter (Colorimeter, PCE-CSM 5, Germany). The color parameters of vinegar were determined as L*, a*, and b*. Color was specified from the type of color parameters L* (darkness–lightness), a* (greenness–redness), and b* (blue–yellowness). The total color difference (Δ*E*), chroma (*C*), and hue angle (*h*) were calculated according to eqs 3–5^60^
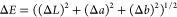
3
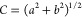
4
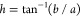
5

### 2.7 Total Soluble Solids, Titratable Acidity, and pH

The total soluble solids (TSS) of vinegar samples were analyzed using a refractometer and were given in Brix (°Bx). pH values were detected using a pH/mV Meter (Hannah, USA). The titratable acidity (TA) was calculated by potentiometric titration of vinegar with NaOH (0.1 N) and was expressed as gram tartaric acid per 100 mL of vinegar sample.^61^

### 2.8 Statistical Analysis

All analyses were performed in triplicate and presented as mean ± standard deviation (SD). Results were evaluated by one-way analysis of variance. Tukey’s HSD test with a significance level of *p* < 0.05 was used to assess differences between means. Data were evaluated by the SPSS 22.0 statistic program (SPSS Inc., USA). Principal component analysis (PCA) was performed by the JMP statistic program (12.2.0 SAS Institute, USA). The Pearson correlation coefficient was analyzed by the OriginPro statistic program (version 2017, OriginLab, USA).

## 3 Results and Discussion

### 3.1 Evaluation of Bioactive Components

The thermal and nonthermal treatments lead to different effects on the phenolic compounds of buckthorn vinegar. The pasteurization process caused a significant decrease in the phenolic content of samples (*p* < 0.05). However, the ultrasonication and the UV treatment caused a significant increase in the phenolic content of the BV samples. In addition, applying a lower dose of UV-C irradiation resulted in higher phenolic content, while longer ultrasonication caused the same effect. The flavonoid content of the BV samples was altered similarly to that of the phenolics, as given in Table 2. The pasteurization process decreased the flavonoid content significantly contrary to the ultrasonication process. The UV-C treatment increased the flavonoid content, but there was no significant difference compared to the control. Similar to the phenolic content, the samples’ flavonoid level and antioxidant activity increased as the sonication time increased. Likewise, the pasteurization caused a significant decrease in the antioxidant activity. Although longer ultrasonication time increased the antioxidant activity significantly, shorter exposure time of UV-C exhibited a similar effect in terms of antioxidant activity. The highest antioxidant activity detected in the most extended ultrasonication treatments was 8.18 ± 0.13 (DPPH) and 9.32 ± 0.13 mg TEAC/mL (CUPRAC). The nonthermal treatments did not significantly affect the BV samples’ ascorbic acid content (Table 2). Besides, the pasteurization process decreased the ascorbic acid level to 2.6 ± 0.02 mg/100 mL compared to the control.

**Table 2:** Effects of Ultrasonication and UV-C Treatment on Some Bioactive Components and Physicochemical Properties of the BV^a^

	total phenolic compound (mg GAE/L)	total flavonoids (mg CE/L)	DPPH (mg TEAC/mL)	CUPRAC (mg TEAC/mL)	ascorbic acid (mg/100 mL)	HMF (mg/L)	pH	TA (g acetic acid /L)	TSS (°Bx)
C-BV	1524.46 ± 5.01^b^	529.39 ± 0.74^bc^	7.39 ± 0.03^bc^	8.57 ± 0.07^b^	2.73 ± 0.02^b^	0.13 ± 0.01^a^	3.41 ± 0.01^b^	3.83 ± 0.01^ab^	4.70 ± 0.00^a^
P-BV	1486.30 ± 8.47^a^	513.59 ± 5.05^a^	6.98 ± 0.25^a^	8.06 ± 0.06^a^	2.6 ± 0.02^a^	0.21 ± 0.02^c^	3.41 ± 0.01^b^	3.81 ± 0.02^a^	4.70 ± 0.00^a^
S1-BV	1533.20 ± 6.72^bc^	540.07 ± 3.32^de^	7.46 ± 0.09^bc^	8.72 ± 0.13^bc^	2.7 ± 0.02^bc^	0.13 ± 0.01^a^	3.40 ± 0.00^ab^	3.84 ± 0.02^abc^	4.77 ± 0.06^a^
S2-BV	1555.70 ± 1.65^d^	542.9 ± 2.79^ef^	7.61 ± 0.14^c^	9.12 ± 0.06^de^	2.65 ± 0.02^ab^	0.14 ± 0.00^a^	3.40 ± 0.00^ab^	3.85 ± 0.02^abc^	4.80 ± 0.00^a^
S3-BV	1586.39 ± 9.74^e^	550.04 ± 3.13^f^	8.18 ± 0.13^d^	9.32 ± 0.13^e^	2.67 ± 0.02^abc^	0.14 ± 0.00^a^	3.40 ± 0.01^ab^	3.88 ± 0.03^c^	4.80 ± 0.00^a^
U1-BV	1517.87 ± 6.65^b^	526.86 ± 3.37^b^	7.1 ± 0.12^ab^	8.03 ± 0.19^a^	2.65 ± 0.02^abc^	0.18 ± 0.01^b^	3.40 ± 0.01^ab^	3.84 ± 0.02^abc^	4.73 ± 0.06^a^
U2-BV	1535.45 ± 6.55^bcd^	532.04 ± 2.74^bcd^	7.42 ± 0.13^bc^	8.75 ± 0.09^bc^	2.69 ± 0.02^bc^	0.18 ± 0.00^b^	3.40 ± 0.00^ab^	3.86 ± 0.02^abc^	4.77 ± 0.06^a^
U3-BV	1553.52 ± 3.32^cd^	535.68 ± 0.92^cde^	7.68 ± 0.11^c^	8.91 ± 0.03^cd^	2.68 ± 0.02^abc^	0.17 ± 0.00^b^	3.39 ± 0.01^a^	3.87 ± 0.01^bc^	4.77 ± 0.06^a^

a ^a–f^ Different letters in each column indicate significant differences between the samples (*p* < 0.05) (*n* = 3 ± SD). C-BV, control BV; P-BV, pasteurized BV; S1-BV, 6 min, 26 kHz, 80 amplitude; S2-BV, 8 min, 26 kHz, 80 amplitude; S3-BV, 10 min, 26 kHz, 80 amplitude; U1-BV, UV-C-treated BV (143.2 mJ cm^–2^); U2-BV, UV-C-treated BV (36.0, mJ cm^–2^); U3-BV, UV-C-treated BV (18.0 mJ cm^–2^).

In correspondence with the results of bioactive compounds, UV-C treatment increased the antioxidant activity of koruk (unripe grape) vinegar^28^ and apple juice.^38^ The ascorbic acid content findings were similar to some studies that reported that UV-C light was ineffective on the ascorbic acid content of orange juice.^37,62^ Moreover, Wang et al. (2020) reported a nonsignificant increase in the ascorbic acid content of the ultrasonication–UV combination of mango juice.^54^ Our results were similar to previous studies on samples exposed to UV-C radiation, which reported induction of flavonoid content.^28,63,64^ Esua et al. (2019) observed a considerable increase in bioactive compound content after UV-C treatment.^22^ The increase in bioactive compounds could be explained by the fact that UV-C has been shown to have positive interactions, indicating an increase in the enzymes responsible for flavonoid and phenolic biosynthesis^65^ such as anthocyanidin synthase, chalcone synthase phenylalanine ammonia-lyase, and stilbene synthase.^66^ Different researchers reported that ultrasound treatments enriched the bioactive components of juice and vinegar samples effectively.^26,67−69^ Yıkmış et al. (2021) reported that the bioactive components of tomato vinegar were enriched with ultrasound treatment and positive effects on health were determined.^70^ Following our results, ultrasound treatment caused an increase in bioactive components, such as total polyphenolic content, and improved antioxidant characteristics of blueberry vinegar. The optimum operation conditions were reported as amplitude and time of 78.50% and 3.96 min, respectively.^71^ The enrichment of bioactive components in ultrasound treatments can be attributed to increasing material release from the cell walls due to cavitation.

### 3.2 Cytotoxicity Assay

**Figure 2:**
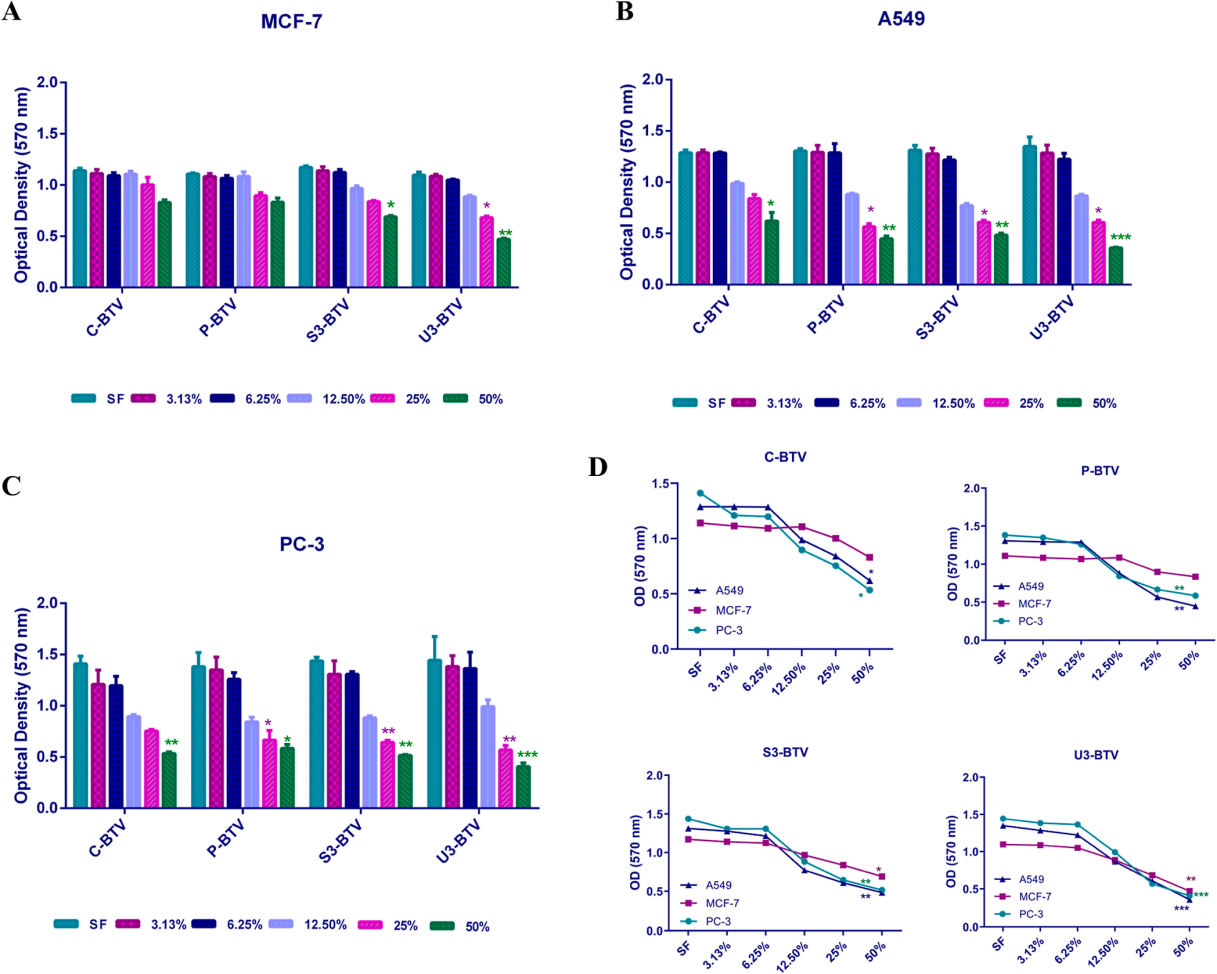
Effect of different concentrations of the BV samples on cell viability in (A) MCF-7 breast cancer, (B) A549 lung cancer, and (C) PC-3 prostate cancer cells. (D) C-BV, P-BV, S3-BV, and U3-BV samples were shown to reduce cell viability in all three different cell lines. *p < 0.05, **p < 0.01, ***p < 0.001.

The cytotoxic effects of U-BV were found to be more significant in MCF7, PC-3, and A549 cells than in the other groups. While no cytotoxic effects of C-BV and P-BV groups were found in MCF-7 cells, the effective concentrations of S3-BV and U3-BV vinegar samples were found to be 56.52 and 40.96%, respectively (Figure 2A). In addition, the effective concentrations of P-BV, S3-BV, and U3-BV samples in A549 cells were found to be 34.14, 32.67, and 29.5%, respectively (Figure 2B). Like lung cancer cells, the effective concentrations of P-BV, S3-BV, and U3-BV samples in prostate cancer cells were 35.28, 31.95, and 29.8%, respectively (Figure 2C). Interestingly, the cytotoxic activity of BV samples treated with UV radiation was more significant than the pasteurization and ultrasound processes (Figure 2D).

In addition to currently applied medical treatments, the use of traditional and alternative treatment approaches is increasing in the fight against cancer. In particular, the use of plant extracts and fermented products, such as vinegar, in supportive treatment draws the attention of researchers in this direction. The first studies in this area can be listed as anticancer, antimicrobial, and antioxidant determination of these products in in vitro experiments. The traditional use of *P. spinosa* in hypertension, diabetes, and gastrointestinal diseases has been previously reported. In addition, *P. spinosa* L. fruit extracts have been reported to have anticarcinogenic activity in various brain and pancreatic cancer cells.^72^ Extracts of *P. spinosa* L. leaves also have a cytotoxic effect on the hepatocellular cancer cell HepG2 and stimulate apoptosis in a dose-dependent manner.^73^ In addition to its antioxidant and anticancer effects, *P. spinosa* L. has wound-healing and antiaging properties.^74^ However, no studies were conducted on the anticancer properties of vinegar obtained from *P. spinosa* L. fruits. In this study, vinegar samples obtained from *P. spinosa* L. fruits by conventional, ultrasound, and UV processes showed cytotoxic effects on lung, breast, and prostate cancer cells. All of these results show that *P. spinosa* L. has a potent anticancer activity. Various herbs and products, such as vinegar and samples produced from plants or their fruits, with synthetic drugs in cancer treatment may be an alternative strategy to create synergistic anticancer effects, reduce individual drug-related toxicity, suppress multidrug-related resistance, and increase therapeutic efficacy.

### 3.3 Physicochemical Properties, HMF Content, and Color Measurements

The pH, TA, TSS, and HMF results of the BV samples are given in Table 2. There were no significant differences in pH values of BV samples compared with C-BV (*p* < 0.05). The treatments except for ultrasonication (S3-BV) showed no significant effect on the TA (g acetic acid/L) of BV samples. There were also no significant differences between the treatments regarding TSS (°Bx). The outcomes demonstrated consistency with the previous research that UV-C treatment did not lead to significant changes in different products.^18,28,75,76^ Similar pH, TA, and TSS results were reported for ultrasound-treated carrot,^77^ strawberry juice,^78^ Kasturi lime juice,^79^ mango juice,^80^ and carrot juice.^81^ In addition to these, Pokhrel and Soria (2017)^82,83^ stated that ultrasonication could maintain the TSS and pH of mango juice. HMF is formed in the Maillard reaction as well as during caramelization as the result of hexose dehydration under high-temperature environments or acidic conditions^84,85^ such as fruit juices and syrups. There was a significant (*p* < 0.05) effect of all treatments except ultrasonication on the HMF content of the BV samples. The pasteurization process caused the maximum increase (0.21 ± 0.02) in the HMF content. UV-C treatment also increased the amount of HMF significantly (*p* < 0.05); however, this was lower than the amount caused by pasteurization. Similarly, it was found that an increase in the HMF content also resulted in an increase in the absorbed dose of irradiation.^84^ Unlikely, the HMF content of UV-C-treated orange juice decreased after a UV-C dose of 68.75 mJ/cm^2^.^39^ In addition to this, it was reported that HMF content of UV-C-treated apple and grape juices was not significantly affected after UV-C treatment.^39^ In the high-intensity ultrasound treatment applied to baobab fruit pulp, no significant change was observed in the HMF value as in our study.^25^

The effects of ultrasonication and UV-C irradiation treatments on the color values of the BV are shown in Table 3. The pasteurization did not cause a significant change in the color values of samples and, unlikely, the other quality parameters; however, some of the ultrasonication and UV-C conditions caused a significant increase in the color values of BV. The ultrasonication and UV-C treatment increased redness, yellowness, and chroma values. All nonthermal treatments except the lowest dose of UV-C caused a nonsignificant effect in brightness and the hue of the BV. Similar results have been reported, indicating that UV-C exposure of mango juice^80^ and high-intensity ultrasound of orange juice^86^ do not considerably change the hue value. It was reported that the b* value decreased after thermosonication in blood fruit (*Haematocarpus validus*) juice,^87^ but on the contrary, an increase was detected in S1-BV and S2-BV samples in our study. Wang et al. (2020) reported that longer treatment of ultrasonication affected the color values of mango juice. This process increases L* value and decreases a* and b* values, so the mango juice seems to be brighter and greener.^54^ The highest increase in Δ*E* was observed in the S2-BV and U3-BV samples compared to the P-BV sample. However, the Δ*E* values can be classified as slightly noticeable (0.5 < Δ*E* < 1.5).^88^ Therefore, the most significant changes in nonthermal treatments fell within the “slightly noticeable” range. According to Birmpa et al. (2013), the lettuce samples exhibited a sharp decrease in brightness as the ultrasonication time increased.^30^ However, in accordance with our findings, ultrasonication for up to 10 min had no discernible impact on the color values of the lettuce and strawberry samples, or samples exposed to lesser doses of UV-C, as compared to the control sample.

**Table 3:** Effects of Ultrasound and UV-C Treatment on the Color Values of the BV Samples^a^

	L*	a*	b*	*C*	*h*	Δ*E*
C-BV	24.93 ± 0.04^ab^	12.27 ± 0.06^ab^	9.58 ± 0.04^a^	15.56 ± 0.07^a^	37.98 ± 0.08^ab^	
P-BV	24.79 ± 0.04^ab^	12.26 ± 0.04^ab^	9.58 ± 0.10^a^	15.56 ± 0.10^a^	38.01 ± 0.20^ab^	0.2 ± 0.04^a^
S1-BV	25.37 ± 0.50^bc^	12.68 ± 0.04^c^	10.23 ± 0.46^b^	16.31 ± 0.27^cd^	38.88 ± 1.25^b^	0.95 ± 0.56^ab^
S2-BV	24.81 ± 0.03^ab^	13.1 ± 0.05^d^	10.13 ± 0.08^b^	16.56 ± 0.09^d^	37.72 ± 0.09^ab^	1.01 ± 0.16^b^
S3-BV	24.88 ± 0.21^ab^	12.55 ± 0.15^bc^	9.53 ± 0.08^a^	15.76 ± 0.07^ab^	37.24 ± 0.54^a^	0.36 ± 0.16^ab^
U1-BV	24.84 ± 0.08^ab^	12.17 ± 0.03^ab^	9.91 ± 0.11^ab^	15.69 ± 0.09^ab^	39.14 ± 0.28^bc^	0.38 ± 0.07^ab^
U2-BV	24.63 ± 0.35^a^	12.68 ± 0.36^c^	10.02 ± 0.14^ab^	16.12 ± 0.35^bcd^	38.32 ± 0.56^ab^	0.71 ± 0.37^ab^
U3-BV	25.67 ± 0.24^c^	12.05 ± 0.07^a^	10.36 ± 0.18^b^	15.89 ± 0.08^abc^	40.67 ± 0.64^c^	1.10 ± 0.26^b^

a ^a–f^ Different letters in each column indicate significant differences between the samples (*p* < 0.05) (*n* = 3 ± SD). C-BV, control BV; P-BV, pasteurized BV; S1-BV, 6 min, 26 kHz, 80 amplitude; S2-BV, 8 min, 26 kHz, 80 amplitude; S3-BV, 10 min, 26 kHz, 80 amplitude; U1-BV, UV-C-treated BV (143.2 mJ cm^–2^); U2-BV, UV-C-treated BV (36.0, mJ cm^–2^); U3-BV, UV-C-treated BV (18.0 mJ cm^–2^).

### 3.4 Volatile Organic Compounds

**Figure 3:**
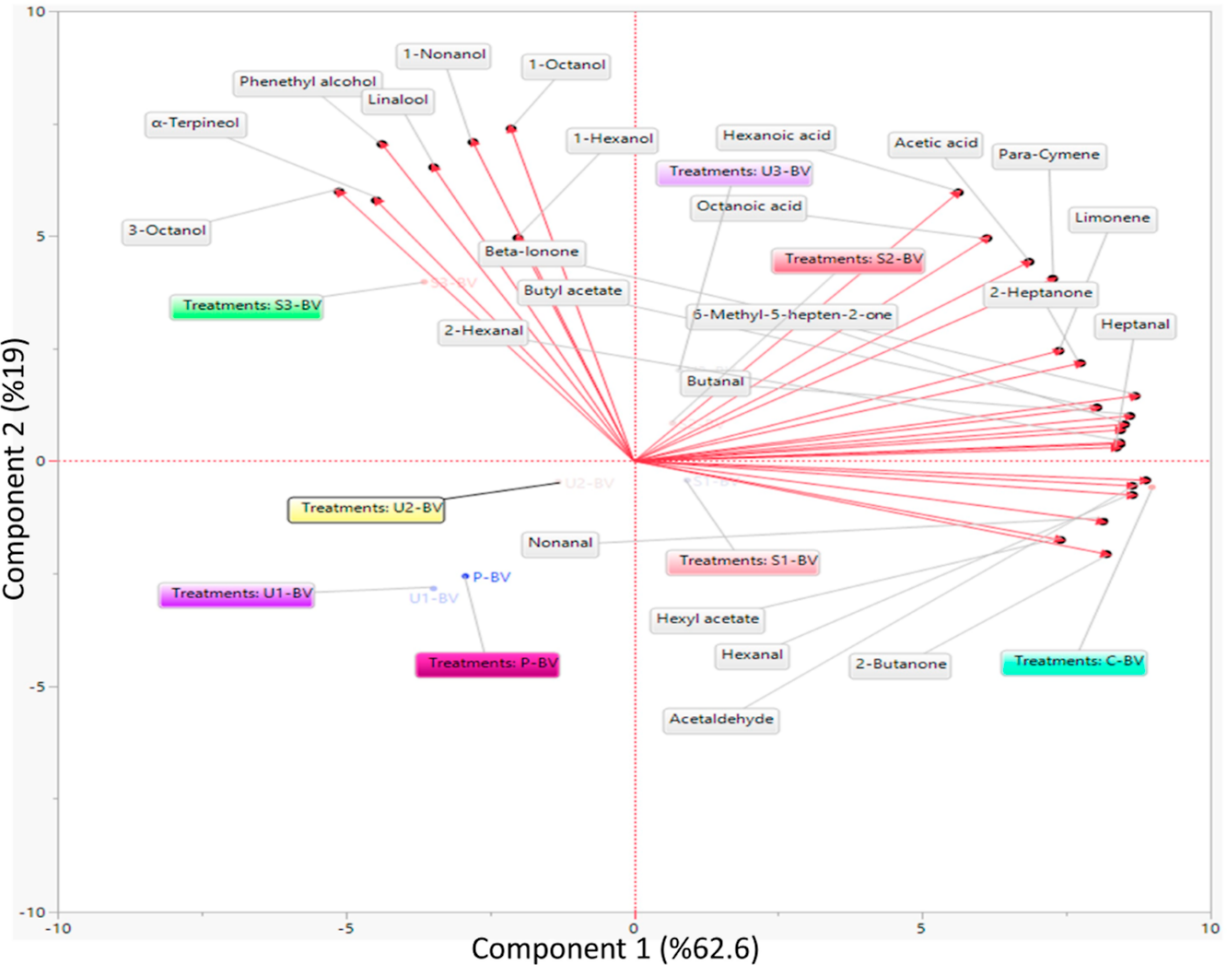
PCA results from volatile organic compounds with the BV.

The identified volatile compounds of the BV samples are given in Table 4. The PCA was used to evaluate the differences between eight BV samples in terms of volatile compounds (Figure 3). The PCA explains the distribution of samples on two principal components. Eigenvector values in the score graph where all vinegar samples were evaluated as PC1 = 62.6% and PC2 = 19%. The PCA is a suitable tool for distinguishing the BV samples and clustering volatile compounds according to their chemical structures. Hexyl acetate, hexanal, acetaldehyde, nonanal, and 2-butanone were in the same cluster with volatile compounds. S3-BV was clustered with 7 volatile compounds. The volatile compounds (12 units) clustered with U3-BV and S2-BV samples among all treated and untreated samples. In addition, U1-BV, U2-BV, and P-BV did not cluster with volatile compounds.

**Table 4:** VOC Profiles of the BV Samples^a^

VOCs	RI	C-BV (μg/kg)	P-BV (μg/kg)	S1-BV (μg/kg)	S2-BV (μg/kg)	S3-BV (μg/kg)	U1-BV (μg/kg)	U2-BV (μg/kg)	U3-BV (μg/kg)
acetaldehyde	823	2.87 ± 0.13^a^	1.89 ± 0.13^b^	2.14 ± 0.15^b^	2.19 ± 0.23^b^	1.79 ± 0.16^b^	1.83 ± 0.12^b^	1.89 ± 0.03^b^	2.08 ± 0.01^b^
methyl acetate	826	0.20 ± 0.08^a^	nd	0.13 ± 0.03^ab^	0.07 ± 0.04^ab^	nd	nd	nd	0.08 ± 0.04^ab^
butanal	830	0.81 ± 0.08^a^	0.56 ± 0.06^ab^	0.62 ± 0.05^ab^	0.61 ± 0.10^ab^	0.52 ± 0.08^b^	0.46 ± 0.04^b^	0.59 ± 0.01^ab^	0.66 ± 0.05^ab^
ethyl acetate	885	0.84 ± 0.10^a^	0.58 ± 0.06^ab^	0.67 ± 0.06^ab^	0.65 ± 0.05^ab^	0.48 ± 0.08^b^	0.48 ± 0.07^b^	0.6 ± 0.03a^b^	0.65 ± 0.12^ab^
2-butanone	904	0.52 ± 0.11^a^	0.34 ± 0.08^a^	0.39 ± 0.07^a^	0.34 ± 0.09^a^	0.27 ± 0.04^a^	0.35 ± 0.04^a^	0.34 ± 0.06^a^	0.42 ± 0.12^a^
butyl acetate	1072	0.31 ± 0.11^a^	0.22 ± 0.01^a^	0.27 ± 0.08^a^	0.26 ± 0.06^a^	0.22 ± 0.07^a^	0.2 ± 0.04^a^	0.27 ± 0.08^a^	0.26 ± 0.08^a^
hexanal	1081	5.62 ± 0.25^a^	3.73 ± 0.14^b^	4.32 ± 0.16^b^	3.97 ± 0.20^b^	3.67 ± 0.18^b^	3.68 ± 0.19^b^	3.77 ± 0.24^b^	3.92 ± 0.14^b^
2-heptanone	1186	0.69 ± 0.10^a^	0.42 ± 0.06^b^	0.54 ± 0.04^ab^	0.58 ± 0.08^ab^	0.49 ± 0.06^ab^	0.46 ± 0.04^ab^	0.39 ± 0.03^b^	0.55 ± 0.09^ab^
heptanal	1190	1.96 ± 0.09^a^	1.36 ± 0.11^b^	1.52 ± 0.18^b^	1.56 ± 0.09^ab^	1.41 ± 0.11^b^	1.38 ± 0.06^b^	1.29 ± 0.01^b^	1.45 ± 0.06^b^
limonene	1198	1.52 ± 0.13^a^	1.29 ± 0.05^a^	1.34 ± 0.18^a^	1.50 ± 0.12^a^	1.32 ± 0.06^a^	1.27 ± 0.08^a^	1.29 ± 0.06^a^	1.4 ± 0.09^a^
2-hexanal	1224	3.92 ± 0.23^a^	2.56 ± 0.18^b^	2.98 ± 0.20^b^	2.60 ± 0.12^b^	2.64 ± 0.15^b^	2.59 ± 0.13^b^	2.69 ± 0.32^b^	3.1 ± 0.21^b^
hexyl acetate	1274	0.68 ± 0.06^a^	0.45 ± 0.05^a^	0.48 ± 0.07^abc^	0.45 ± 0.09^abc^	0.27 ± 0.00^c^	0.37 ± 0.06^bc^	0.54 ± 0.04^abc^	0.59 ± 0.08^ab^
*para*-cymene	1278	3.49 ± 0.27^a^	2.34 ± 0.16^b^	2.78 ± 0.17^b^	2.75 ± 0.28^b^	2.96 ± 0.09^ab^	2.41 ± 0.07^b^	2.40 ± 0.06^b^	2.82 ± 0.20^ab^
6-methyl-5-hepten-2-one	1346	1.22 ± 0.18^a^	0.75 ± 0.09^ab^	0.99 ± 0.15^ab^	0.85 ± 0.11^ab^	0.69 ± 0.01^b^	0.57 ± 0.11^b^	0.79 ± 0.01^ab^	0.91 ± 0.11^ab^
1-hexanol	1358	0.12 ± 0.01^c^	0.19 ± 0.04^bc^	0.20 ± 0.06^bc^	0.28 ± 0.06^bc^	0.36 ± 0.13^bc^	0.2 ± 0.06^bc^	0.47 ± 0.13^ab^	0.68 ± 0.06^a^
3-octanol	1395	0.25 ± 0.04^c^	0.36 ± 0.09^bc^	0.52 ± 0.11^abc^	0.47 ± 0.12^abc^	0.75 ± 0.13^a^	0.42 ± 0.04^abc^	0.67 ± 0.08^ab^	0.55 ± 0.09^abc^
nonanal	1398	5.29 ± 0.28^a^	3.69 ± 0.25^b^	4.23 ± 0.19^b^	4.05 ± 0.30^b^	3.58 ± 0.22^b^	4.04 ± 0.18^b^	4.45 ± 0.24^ab^	4.37 ± 0.13^b^
acetic acid	1471	0.51 ± 0.16^a^	0.29 ± 0.08^a^	0.43 ± 0.05^a^	0.49 ± 0.11^a^	0.38 ± 0.08^a^	0.23 ± 0.05^a^	0.40 ± 0.08^a^	0.43 ± 0.10^a^
benzaldehyde	1541	3.59 ± 0.23^a^	2.56 ± 0.26^b^	3.06 ± 0.23^ab^	3.11 ± 0.12^ab^	2.62 ± 0.23^ab^	2.62 ± 0.18^b^	3.07 ± 0.12^ab^	3.02 ± 0.11^ab^
linalool	1548	0.58 ± 0.08^c^	0.81 ± 0.06^abc^	1.02 ± 0.10^abc^	1.13 ± 0.16^ab^	1.26 ± 0.11^a^	0.74 ± 0.12^ab^	0.65 ± 0.10^ab^	1.01 ± 0.20^abc^
1-octanol	1564	0.32 ± 0.09^b^	0.24 ± 0.03^b^	0.23 ± 0.04^b^	0.45 ± 0.05^ab^	0.58 ± 0.09^a^	0.34 ± 0.03^ab^	0.41 ± 0.06^ab^	0.44 ± 0.07^ab^
1-nonanol	1662	nd	nd	0.10 ± 0.04^bc^	nd	0.23 ± 0.06^a^	nd	0.13 ± 0.03^ab^	0.15 ± 0.04^ab^
α-terpineol	1694	nd	0.29 ± 0.04^bc^	0.19 ± 0.04^bc^	0.33 ± 0.08^ab^	0.53 ± 0.16^a^	0.14 ± 0.04^abc^	nd	0.34 ± 0.08^ab^
hexanoic acid	1854	0.40 ± 0.08^a^	0.18 ± 0.03^a^	0.26 ± 0.04^a^	0.31 ± 0.08^a^	0.36 ± 0.10^a^	0.17 ± 0.04^a^	0.30 ± 0.06^a^	0.28 ± 0.06^a^
phenethyl alcohol	1925	0.28 ± 0.04^b^	0.37 ± 0.03^ab^	0.41 ± 0.09^ab^	0.35 ± 0.06^ab^	0.54 ± 0.10^a^	0.32 ± 0.06^ab^	0.38 ± 0.04^ab^	0.47 ± 0.06^ab^
β-ionone	1968	3.38 ± 0.20^a^	2.22 ± 0.18^b^	2.68 ± 0.27^ab^	2.62 ± 0.10^ab^	2.44 ± 0.13^b^	2.36 ± 0.24^b^	2.58 ± 0.09^b^	2.77 ± 0.29^ab^
octanoic acid	2067	0.10 ± 0.02^a^	nd	nd	0.04 ± 0.02^ab^	0.06 ± 0.03^ab^	nd	nd	0.06 ± 0.04^ab^

Total (mg/kg)								
esters	2.03	1.25	1.55	1.43	0.97	1.05	1.41	1.58
alcohols	1.55	2.26	2.67	3.01	4.25	2.16	2.71	3.64
aldehydes	24.06	16.35	18.87	18.09	16.23	16.60	17.75	18.6
ketones	5.81	3.73	4.6	4.39	3.89	3.74	4.1	4.65
acids	1.01	0.47	0.69	0.84	0.8	0.40	0.7	0.77
terpenes	5.01	3.63	4.12	4.25	4.28	3.68	3.69	4.22

a ^a–c^ Different letters in each row indicate significant differences between the samples (*p* < 0.05) (*n* = 3 ± SD). C-BV, control BV; P-BV, pasteurized BV; S1-BV, 6 min, 26 kHz, 80 amplitude; S2-BV, 8 min, 26 kHz, 80 amplitude; S3-BV, 10 min, 26 kHz, 80 amplitude; U1-BV, UV-C-treated BV (143.2 mJ cm^–2^); U2-BV, UV-C-treated BV (36.0, mJ cm^–2^); U3-BV, UV-C-treated BV (18.0 mJ cm^–2^); nd, not detected; RI, retention index.

The BV samples showed 25–27 VOCs (Table 4), and the most identified groups were aldehydes, (7) alcohols, (7) esters, and (4) ketones (4). The lowest VOCs were found in U1-BV and P-BV at 27.63 and 27.69 μg/kg, respectively. The highest content of VOCs was found in the U3-BV and S1-BV samples at 33.46 and 32.50 μg/kg, respectively. The most minor difference was detected in U3-BV at 6.01 μg/kg compared to C-BV. Considering the overall effects, it was determined that pasteurization significantly affected the VOC profile of the BV. Ultrasonication and UV-C treatments were found to maintain the profile better, and U3-BV was found to be successful with the most minor reduction. Aldehydes were the predominant organic volatiles after UV-C, ultrasound, and pasteurization treatments. Among the aldehydes identified in the BV, benzaldehyde represented the most significant portion of total quantified aldehydes. Besides, C-BV showed the highest aldehyde content. All treatments caused a reduction in the total amount of aldehydes, ketones, acids, and terpenes. However, an increase has been observed in the alcohols of all treated samples. The highest increase was obtained in the 10 min ultrasonication. Linalool (responsible for floral, citrus, and fruit volatile compounds) increased in all samples compared to the C-BV sample. The S3-BV sample showed the largest increase, 0.68 μg/kg. In addition, the treatments had no significant effect on limonene. Similar to our results, Kumar Gupta et al. (2021) reported an increase in linalool by sonication. On the other hand, all treatments decreased the content of terpenes. Similar to these results, Wang et al. (2020) reported a decrease in mango juice terpenes after ultrasonication and UV treatment, which could be due to the intense acoustic cavitation of high-intensity ultrasound. Significant differences were observed in 1-octanol at 10 min ultrasonicated BV samples and 3-octanol in ultrasonication and UV-C treatments (*p* < 0.05). In correspondence with these results, an increase in octanal content was seen after the ultrasonication process that led to the removal of bitterness in citrus fruit juice in a previous work.^89^ Similarly, Yıkmış et al. (2021)^90^ reported that ultrasonication maintained the VOCs better than the pasteurization process. Changes in VOCs in ultrasonication treatment can be attributed to the strong cavitation effect of ultrasound.^54,89,91^

## 4 Conclusions

This study compared the effectiveness of two nonthermal disinfection techniques, ultrasonication and UV-C, on their ability to influence the quality characteristic and bioactive attributes of the traditionally produced BV. The flavor compounds, including linalool, octanal, and terpineol, were enhanced by ultrasonication and UV-C treatment compared to the control. Nonthermal technologies did not cause significant changes in the physicochemical properties and HMF content of BV samples. Due to its high anticarcinogenic activity, the UV-C-treated BV, rich in bioactive components, can be a supplementary agent for cancer patients. As a result, ultrasound and UV-C processes enhanced the quality of the BV, and this study may provide a basis for further studies on the quality and health effects.
